# Measurement of Dehumanization, Self-Dehumanization, and Empathy as Mediating Factors Among Healthcare Professionals

**DOI:** 10.3390/healthcare13010075

**Published:** 2025-01-03

**Authors:** Aikaterini Roupa, Athina Patelarou, Konstantinos Giakoumidakis, Kyriaki Fousiani, Marianna Miliaraki, Eirini Stratidaki, Evridiki Patelarou

**Affiliations:** 1 Department of Nursing, School of Health Sciences, Hellenic Mediterranean University, 71410 Heraklion, Greecekongiakoumidakis@hmu.gr (K.G.); epatelarou@hmu.gr (E.P.); 2 Department of Psychology, University of Groningen, 9712 Groningen, The Netherlands; k.fousiani@rug.nl; 3 Pediatric Intensive Care Unit, School of Medicine, University of Crete, 70013 Heraklion, Greece; med1p1130027@med.uoc.gr

**Keywords:** dehumanization, hetero-dehumanization, healthcare professionals, empathy, personality

## Abstract

Background: Dehumanization refers to the tendency of individuals or groups to attribute fewer human characteristics to other individuals or groups (referred to as hetero-dehumanization) or to themselves (referred to as self-dehumanization). This phenomenon currently seems to predominate in the medical and nursing professions. Indeed, healthcare environments facilitate latent forms of dehumanization due to their structure, organization, and inherent professional demands. This study aimed to investigate the association between hetero- or self-dehumanization and personality traits of healthcare professionals, as well as the possible key mediating role of empathy in this relationship. Methods: A total of 1150 healthcare employees were recruited for the current study with a mean age of 45.13 years. Data were collected through a questionnaire completed by health professionals. Results: Statistically significant relationships were found between self- and hetero-dehumanization and most personality traits (extraversion, agreeableness, conscientiousness). A mediation analysis revealed that empathy mediates the effects of personality traits on hetero- and self-dehumanization. Conclusions: The present study addresses the vital role of personality traits of healthcare professionals on dehumanizing oneself or patients, offering insights into improving therapeutic relationships through the cultivation of empathy.

## 1. Introduction

Dehumanization is a complex social phenomenon referring to the process in which people are stripped of characteristics that make them human either by other people (hetero-dehumanization) or by themselves (self-dehumanization) [1]. According to Haslam (2006), hetero-dehumanization encompasses [2] two primary forms: animalistic dehumanization and mechanistic dehumanization. Animalistic dehumanization is defined as the attribution of non-human characteristics to others, treating them as “animals” or children, without social norms, emotions, or self-control. This often leads to the withholding of prosocial feelings or empathy and results in humiliation and contempt towards other human beings. This phenomenon has been attributed to increased activity in the inferior frontal gyrus and is linked to a higher risk of hostility and violence [2,3,4,5,6]. Conversely, mechanistic dehumanization is characterized by the denial of human traits of other people, making individuals appear cold, passive, and devoid of will or mentality [2,7,8,9]. Thus, mechanistic dehumanization involves objectifying others as if they were akin to inanimate mechanical systems, leading to feelings and actions of indifference [5,6,10].

Self-dehumanization is a phenomenon where individuals dehumanize themselves, using this mechanism as a coping strategy to protect themselves from emotional pain or to conform to societal demands. Self-dehumanization is linked to feelings of helplessness, an inability to express personal capabilities and intelligence, or a focus on primary goals, often resulting in the deprivation of kindness and moral characteristics [11,12,13]. While self-dehumanization is frequently associated with avoidance of self-awareness, low self-esteem, a lack of meaning in life, and the experience of intense negative emotions (such as shame, sadness, anger, and guilt), individuals may adopt such processes to reduce the psychological burden of witnessing others’ suffering [14].

### 1.1. Dehumanization in Healthcare

Mechanistic dehumanization currently predominates in the healthcare sector, particularly in hospitals [2]. Based on biomedical models, modern medicine primarily focuses on physical disorders, often neglecting the individualized needs and psychological care of patients. As a result, the human body is treated as a “machine,” and illness is viewed as a mere malfunction [15]. Physicians and nurses, due to time constraints, struggle to provide personalized care, which often undermines trust in their relationships with patients [16,17].

Nevertheless, animalistic dehumanization is also common in healthcare. Lebowitz and Ahn (2014) demonstrated that doctors who focus on biological explanations of mental disorders tend to see their patients as fundamentally different from healthy individuals, potentially reinforcing negative social attitudes and dehumanization [18]. Trifiletti et al. (2014) argue that dehumanizing patients by attributing animal-like traits to them is one way nurses manage stress and the associated symptoms stemming from their work [19]. Healthcare professionals may attribute fewer human characteristics to patients, possibly as a means of protecting themselves from burnout [20]. However, the lack of empathy accompanying this approach can negatively affect the quality of care provided [21].

Finally, self-dehumanization is observed when healthcare workers are exposed to continuous work stress, as is common in the healthcare field [22]. In such cases, workers tend to dehumanize themselves, using this strategy as a defense mechanism to shield themselves from negative emotions [23]. This phenomenon is often linked to the perception of patients as not being independent individuals but rather objects [24].

### 1.2. Personality and Dehumanization

Personality traits influence how individuals perceive and interact with others, especially within healthcare settings. Traits such as agreeableness, conscientiousness, and openness can promote empathy and a more humanizing treatment of oneself and others, while other personality tendencies, such as emotional instability, may contribute to less humanizing tendencies [25]. Exploring the role of personality in dehumanization in healthcare in particular may offer valuable insights for fostering compassionate and patient-centered care.

The Big Five personality traits, also known as the Five-Factor Model [26], provide a comprehensive framework for understanding individual differences in personality [27]. These traits are extraversion, agreeableness, conscientiousness, emotional stability (or its opposite, neuroticism), and openness to experience. Extraversion reflects sociability, assertiveness, and a tendency toward positive emotions, influencing how individuals engage with the external world. Agreeableness captures traits such as kindness, and a cooperative nature, shaping interpersonal relationships and conflict resolution. Conscientiousness involves self-discipline, organization, and a preference for planned rather than spontaneous behavior, often associated with goal-oriented actions and reliability. Emotional stability (or the absence of neuroticism), indicates the degree to which individuals experience emotional resilience, calmness, and steadiness versus anxiety, moodiness, and vulnerability. Finally, openness to experience reflects intellectual curiosity, creativity, and a willingness to explore new ideas and experiences. Together, these five dimensions provide a robust model for understanding how personality influences behavior, attitudes, and interactions in various contexts [26,28,29].

Previous research has explored the relationship between personality traits and dehumanization, highlighting a strong association between neuroticism (low emotional stability) or extraversion and hetero-dehumanization [25]. However, studies have yet to examine how healthcare professionals’ personality traits specifically relate to both forms of hetero-dehumanization—animalistic and mechanistic. Additionally, the link between healthcare professionals’ personality traits and self-dehumanization remains largely underexplored. This research seeks to address these gaps in the literature [25].

#### 1.2.1. Personality as Predator of Hetero-Dehumanization

The Big Five personality traits provide a valuable framework for understanding tendencies toward animalistic and mechanistic hetero-dehumanization among healthcare professionals. Animalistic dehumanization, which involves perceiving others as primitive or lacking refinement [2], is likely to be negatively related to agreeableness, extraversion, conscientiousness, openness, and emotional stability. These traits are associated with empathy, perspective-taking, and the ability to recognize the complexity and individuality of others, which may reduce perceptions of patients as primitive, less evolved, or instinct-driven. Conversely, low emotional stability (or high neuroticism) may be linked to greater tendencies toward animalistic dehumanization due to difficulties managing stress and interpersonal challenges. Similarly, mechanistic dehumanization, which entails perceiving others as emotionless or machine-like entities [2], may also be negatively associated with agreeableness, conscientiousness, openness, extraversion, and emotional stability. Healthcare professionals with higher levels of conscientiousness may be more attentive to patients’ individual needs, while those with higher agreeableness and extraversion may foster interpersonal warmth and relational connectedness, counteracting mechanistic views. Emotional stability may further mitigate mechanistic dehumanization of patients by promoting calmness and resilience in demanding healthcare environments [25,26].

#### 1.2.2. Personality as Predictor of Self-Dehumanization

In addition to investigating how personality traits influence the dehumanization of patients, it is particularly equally important to explore how personality traits influence self-dehumanization among healthcare professionals. Although this phenomenon has received limited attention in the existing literature [11,12], it warrants closer investigation due to its significant consequences for both the individual and the quality of care provided. Self-dehumanization, which involves viewing oneself as lacking essential human qualities, can impact a professionals’ well-being, job satisfaction, and overall effectiveness in their roles. Given the demanding nature of healthcare environments, understanding how personality traits shape this process could provide valuable insights into mitigating its negative effects and supporting professionals’ mental health and professional efficacy [11].

Building on the above, we argue that emotional stability is expected to negatively relate to self-dehumanization by fostering feelings of adequacy and self-worth. Similarly, high conscientiousness may be negatively linked to self-dehumanization by reinforcing perceptions of personal competence and alignment with societal or professional standards. High extraversion, marked by social engagement and positive interpersonal interactions, is hypothesized to be negatively associated with self-dehumanization by enhancing feelings of belonging and providing positive reinforcement, which can affirm one’s sense of self-worth and humanity.

### 1.3. The Mediating Role of Empathy

The denial of humanity towards others is associated with low empathy for others, while empathy is considered a key prerequisite for overcoming dehumanization [30]. Furthermore, empathy as a dispositional trait is linked to low attribution of humanity to bullies and high attribution of humanity to victims [31]. Empathy is a critical factor in healthcare professionals’ interactions with patients and can significantly shape their perceptions of other people and themselves. Accordingly, in addition to the direct effects of personality traits on others and self-dehumanization, we hypothesize that empathy may play a mediating role in these relationships. Empathy is defined as the ability to understand and share the feelings of others [32] and is strongly linked to the perception of a fully human nature of patients, thereby reducing dehumanization tendencies [13]. Specifically, traits such as agreeableness, emotional stability, and conscientiousness are likely to foster empathy, which, in turn, could reduce both animalistic and mechanistic dehumanization. High agreeableness, which encompasses empathy and kindness [26], may enhance healthcare professionals’ capacity to connect with patients on an emotional level, thus mitigating dehumanization. Similarly, emotional stability (low neuroticism) contributes to emotional resilience [33,34] which could facilitate the ability to empathize even in stressful healthcare environments, lowering, in turn, the development of dehumanizing attitudes toward patients [29]. Conscientious individuals, who value professionalism and responsibility, are likely to demonstrate heightened empathy toward patients, ensuring that they treat patients with dignity and respect, thus lowering dehumanization. In addition, empathy may serve as a buffer against self-dehumanization, with high levels of empathy enabling healthcare professionals to view themselves with greater compassion and self-worth and reducing the internalization of negative feelings, such as that of inadequacy.

### 1.4. Professional Quality of Life

Professional quality of life in the field of nursing and medical care refers to the experience of the worker during the performance of their duties as they provide assistance to patients [35]. It is natural that both the positive and negative aspects of work influence this quality of life. The main dimensions include a positive aspect, which is associated with satisfaction derived from compassion toward patients, and a negative aspect, related to compassion fatigue. Compassion fatigue is comprised of two components: (a) feelings such as exhaustion, frustration, anger, and depression, which are characteristics of professional burnout, and (b) secondary traumatic stress, a negative emotion stemming from fear and work-related trauma [36].

Certain personal characteristics appear to increase the risk of developing professional burnout. These include a strong tendency towards self-criticism, the adoption of dysfunctional coping strategies, chronic sleep deprivation, and a lack of balance between professional and personal life [37]. Additionally, traits such as idealism, perfectionism, and excessive commitment are also associated with professional burnout and are often observed in highly efficient and productive workers. Certain personality types, such as individuals with high levels of neuroticism, are more prone to experiencing burnout. In contrast, extroverted, conscientious, and agreeable individuals are less likely to exhibit such symptoms [38].

### 1.5. The Evaluation of Care Quality by Healthcare Professionals in the Hospital

One of the most significant factors contributing to a nurse’s satisfaction is the quality of the work environment and staffing levels. A well-organized work environment and adequate staffing are directly linked to higher levels of nurse satisfaction. Nurses working in hospitals with more supportive work environments report lower levels of burnout, reduced job dissatisfaction, and a decreased intention to leave their positions [34,39,40].

### 1.6. Purpose of the Study

Dehumanization is a complex phenomenon influenced by various factors, including personality traits, empathy, and working conditions [20,41,42]. Developing patient-centered approaches that consider both physical and psychological needs is vital for improving the quality of care provided. It is not a widely recognized issue in healthcare, since the literature suggests that studies on this field are limited [43,44,45]. This study aimed to investigate the phenomena of hetero-dehumanization and self-dehumanization among healthcare professionals in public hospitals. Additionally, it explored personality traits of healthcare professionals as potential antecedents of both types of dehumanization and empathy as a possible underlying mechanism driving these relationships. By addressing this literature gap in the early recognition of dehumanization, we promote a more holistic approach to patient care, improve the doctor-patient relationship, and enhance the quality of healthcare services. Thus, the following hypotheses were formulated:

**H1:**  *Healthcare professionals’ extraversion, openness to experience, conscientiousness, emotional stability, and agreeableness are negatively associated with (a) mechanistic, and (b) animalistic dehumanization of patients, and (c) self-dehumanization*.

**H2:**  *Empathy mediated the relationship between healthcare professionals’ extraversion, openness to experience, conscientiousness, emotional stability, and agreeableness on the one hand, and (a) mechanistic dehumanization, (b) animalistic dehumanization, and (c) self-dehumanization*.

In addition to the main hypotheses, professional quality of life and the assessment of the quality of care provided by healthcare professionals were additionally measured for exploratory purposes. These variables were included to better understand how broader aspects of professional well-being, such as job satisfaction and emotional resilience, may relate to dehumanization and self-dehumanization. By examining these variables, the study further explores potential links between healthcare professionals’ quality of life and their perceptions and treatment of patients, as well as how their self-perceptions may influence the quality of care provided in hospital settings.

## 2. Materials and Methods

### 2.1. Sampling—Participants

The research was conducted in 14 public hospitals across cities in Greece from March 2022 to September 2022 with a sample of 1150 healthcare workers, including both medical and nursing staff. Hospital departments were categorized into two types: “units” referring to closed departments, such as intensive care units (ICUs), and “wards” representing open clinical departments, like pediatrics or internal medicine. Regarding working hours, “cyclical hours” indicate rotating shift schedules, including early, late, and night shifts, while “shifts” referred specifically to non-rotating work schedules. “Convenience sampling” was used to select the sample, and participants were informed in detail about the purpose and objectives of the study, meeting the predefined inclusion and exclusion criteria, which were professional experience of less than six months and non-desire to participate in the study (Table 1).

### 2.2. Questionnaires

One of the researchers, the same researcher each time, distributed questionnaires to the departments of the participating hospitals. Detailed information about the study’s purpose and objectives was provided to them. The questionnaires were accompanied by a letter providing information about the study, ensuring anonymity, and emphasizing the voluntary nature of participation. Participants were asked to provide certain socio-demographic information, including some work-related details. The average time to complete the questionnaire was 20 min.

In particular, the following questionnaires were administered:

1. Dehumanization Scale (DS): This scale measures hetero-dehumanization and consists of 12 items divided into two subscales: animalistic dehumanization and mechanistic dehumanization. It assesses the extent to which individuals dehumanize other people by denying their human qualities [8]. The items were assessed using a 7-point Likert scale, ranging from 1 (completely disagree) to 7 (completely agree). The responses to statements 2, 4, 6, 7, 9, 10, and 12 are reverse-scored to ensure that all 12 statements are aligned in the same conceptual direction. Research on the coefficient validity ratio has indicated that all items (100%) were acceptable. Each measure has demonstrated a reliability coefficient of 0.86 [8,13].

2. Mechanistic Self-Dehumanization Scale (MSDS): This scale measures self-dehumanization, referring to the dehumanization of oneself. It includes 10 items and evaluates the degree to which individuals perceive themselves as lacking human qualities. The items were measured on a 9-point Likert scale (1 = absolutely disagree to 9 = absolutely agree). The responses to statements 5, 7, 8, and 10 are reverse-scored to ensure that all 10 statements align in the same conceptual direction. The self-dehumanization scale was a unidimensional measure of mechanistic dehumanization, with reports indicating its validity, based on a Cronbach’s alpha of around 0.97 [8,11,12].

3. The Ten-Item Personality Inventory (TIPI; Gosling, Rentfrow, and Swann, 2003) was used to assess participants’ personality traits based on the Big Five dimensions. Participants rated their agreement with 10 items on a 7-point Likert scale ranging from 1 (“Disagree strongly”) to 7 (“Agree strongly”). Each dimension was measured using two items, one positively worded and one negatively worded, which was reverse-scored: Extraversion (e.g., “Extraverted, enthusiastic”), Agreeableness (e.g., “Sympathetic, warm”), Conscientiousness (e.g., “Dependable, self-disciplined”), Emotional Stability (e.g., “Calm, emotionally stable”), and Openness to Experience (e.g., “Open to new experiences, complex”). Reverse-scored items were adjusted before computing scores for each dimension. In this questionnaire, the authors have not based their research on the Cronbach’s Alpha index calculation as it was not among their original objectives. Moreover, each scale consists of only two items, which inherently leads to low reliability indices regardless of the research conditions [46,47].

4. Toronto Empathy Questionnaire (TEQ): This questionnaire assesses emotional empathy and consists of 16 closed-ended questions with predefined answer options, covering a broad range of empathy-related characteristics. It examines individuals’ ability to understand the emotions of other people experiencing distress. This type of empathy is associated with concern for other human beings, self-sacrifice, and altruism. It comprises 16 items, based on a 5-point Likert scale: ranging from 0 (“Never”) to 4 (“Always”). The questionnaire encompasses a wide range of traits commonly associated with the emotional aspect of empathy. Eight items (specifically, 2, 4, 7, 10, 11, 12, 14, and 15) are reverse-scored, meaning their responses must be inverted (e.g., 4 becomes 0, 3 becomes 1, and so on). The TEQ focuses on emotional dimensions, such as responsiveness and concern for other people. The internal consistency of the questionnaire was evaluated using Cronbach’s α. A reliability coefficient of 0.70 or higher was deemed acceptable [48].

5. Professional Quality of Life Scale (ProQOL-CSF-R-IV): The PROQOL consists of 30 questions and is divided into three independent subscales: compassion satisfaction, burnout, and compassion fatigue/secondary traumatic stress. Each subscale is psycho-metrically distinct and cannot be combined with the other subscales. The scoring is based on a 6-point Likert scale, ranging from 0 (“Never”) to 5 (“Very Often”). Regarding reliability, Cronbach’s alpha coefficients for the subscales are as follows: Original scale: compassion satisfaction (alpha = 0.87), burnout (alpha = 0.90), and compassion fatigue (alpha = 0.87), and the Greek version of the ProQOL: compassion satisfaction (alpha = 0.89), burnout (alpha = 0.94), and compassion fatigue (alpha = 0.92) [49,50].

6. Evaluation and Self-Report Questionnaire for the Quality of provided healthcare services: This questionnaire comprises 25 questions categorized into five distinct domains: quality of interaction with patients, immediate patient perception of quality, long-term usefulness of the service, satisfaction, and overall image. It evaluates various dimensions of service quality, including reliability, responsiveness, assurance, and empathy, focusing on the human and psychological aspects of patient care. Reliability analysis of the data were conducted based on Cronbach’s Alpha criterion, followed by the examination of variable correlations (Cronbach’s Alpha = 0.910) [51].

The research was conducted in full compliance with the General Data Protection Regulation (GDPR) [EU 2016/679], effective since 25 May 2018, concerning the protection of sensitive personal data. All necessary approvals were obtained from relevant authorities before the commencement of the study. The data collected were anonymous and used exclusively for research purposes, with access limited to the principal investigator.

Participants were fully informed about the study’s purposes and procedures and provided written consent, with clear notification that their participation was anonymous, their data would be used solely for research purposes, and they retained the right to leave the study at any time without consequence.

## 3. Results

A total of 1150 healthcare personnel were recruited for the current study with a mean age of 45.13 years, meeting the predefined inclusion and exclusion criteria (Table 1). The majority of the sample consisted of females (76.1%), and resided in big cities (53.7%), small towns (32.2%), or villages (14.1%). Specifically, the working cities declared were Athens (15.6%), Thessaloniki (16.0%), Heraklion (30.1%), or other smaller cities (38.4%).

More than half of the participants (56.7%) were declared as married and a significant portion (84.5%) indicated a low annual family income of less than 20,000€. Seven out of ten participants were employed as nurses (71.0%) and a notable percentage (76.9%) had a Master’s degree. Concerning their work departments, the majority reported working in closed departments (51.2%), with a similar percentage (48.7%) employed in open departments. In response to questions about their work experience, the majority stated having between 2 and 10 years in their roles (26.6%) and in their current departments (41.6%).

As healthcare personnel in public hospitals, most participants worked in circular timetables (76.3%), while 23.7% stated working only in morning and afternoon shifts. Participants’ relationship with colleagues was characterized as Very Good or Good (91.6%), and similarly characterized their relationship with patients as Very Good or Good (93.6%). Notably, nearly 2 out of 10 healthcare personnel declared attending psychotherapy sessions (17.8%).

**Figure 1:**
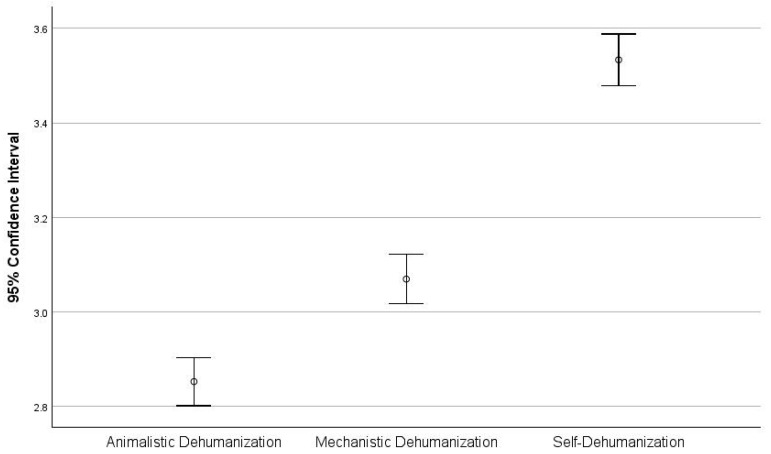
Error bar plot showing the Hetero- Dehumanization Scale and Self-dehumanization scale.

The mean score of the Mechanistic Dehumanization subscale was calculated as 3.07 ± 0.90, and the mean score of the Animalistic Dehumanization subscale was calculated as 2.85 ± 0.88 (Figure 1). The mean score of the Mechanistic Self-Dehumanization scale was calculated as 3.53 ± 0.94 (Figure 1). The internal consistency of questionnaires for each of these types of dehumanization was measured through Cronbach’s alpha, which was 0.646, 0.718, and 0.706, respectively. Cronbach’s alpha coefficient for TEQ was found to be 0.729, and the internal consistency estimates for PROQOL revealed alphas in the range of 0.701–0.86 and the Evaluation and Self-Report Questionnaire for the Quality of provided healthcare services revealed alphas in the range of 0.766–0.865. However, the TIPI questionnaire revealed alphas in the range of 0.33–0.428.

Correlation coefficients between the two dehumanization scales (hetero- and self- dehumanization) and other investigated scales are presented in Table 2. With the exception of the extraversion personality trait, statistically significant negative correlations were found between all personality traits of TIPI and hetero-dehumanization, or self-dehumanization (*p* < 0.001 for all correlations). With regard to the Toronto Empathy Questionnaire, statistically significant correlations were also recorded with all forms of dehumanization (*p* < 0.001). Moreover, the compassion parameter of PROQOL had statistically significant negative correlation, but burnout and secondary traumatic stress showed positive correlations with all forms of dehumanization (*p* < 0.01 for all correlations). It should be noted that all of the above associations have reached statistical significance, although their quantitative relevance is relative, based on Pearson’s correlation coefficients. Non-significant correlations were revealed between age and animalistic, mechanistic, and self-dehumanization (r = −0.033, *p* = 0.258; r = −0.033, *p* = 0.269; r = 0.034, *p* = 0.249, respectively).

The demographic and other characteristic differences are outlined in Table 3. Statistically significant sex differences in relation to self-dehumanization were recorded (*p* = 0.031). More specifically, males reported higher levels of self-dehumanization (M ± SD = 3.64 ± 0.93) compared to women (M ± SD = 3.50 ± 0.94). However, non-significant correlations were observed between sexes and both forms of hetero-dehumanization. Additionally, significant differences were found between the pattern of working hours and mechanistic dehumanization (*p* = 0.017). Healthcare personnel with circular timetables exhibited higher levels of mechanistic dehumanization (M ± SD = 3.10 ± 0.91) compared to those with morning/afternoon shifts (M ± SD = 2.96 ± 0.87). Finally, healthcare workers attending psychotherapy sessions seemed to have a higher tendency towards mechanistic dehumanization, although this parameter did not reach statistical significance.

To examine the hypothesis that empathy acts as a mediator in the relationship between the personality traits of the TIPI scale and both hetero-dehumanization and self-dehumanization scales, mediation analyses were conducted. Based on the findings shown in Table 4, empathy was recorded as a partial mediator of the differential effect of agreeableness or emotional stability on animalistic hetero-dehumanization. However, in cases of health professionals showing traits of extraversion, conscientiousness and openness to experiences, empathy fully mediated the effect against animalistic dehumanization; moreover, for participants showing traits of openness to experiences, empathy was also found to fully mediate the effect against mechanistic dehumanization. Finally, empathy was recorded as a partial mediator in nearly all other mediation analyses regarding mechanistic and self-dehumanization.

## 4. Discussion

Dehumanization is highly relevant to several important psychosocial aspects of medical care, negatively impacting the quality of care and person-centered health services. A recent study validated two measures for assessing both animalistic and mechanistic dehumanization of patients, as well as self-dehumanization by healthcare professionals [8]. Building on this, our current research provides significant evidence that both types of dehumanization affect healthcare professionals to a moderate degree and offers valuable insights into the various mediating factors of dehumanization in healthcare. In fact, there is a growing body of evidence indicating that dehumanization still remains a thorn in healthcare, reflecting intergroup hostility [52]. Dehumanization also has traumatizing influences on certain “fragile” groups, such as the recipients of healthcare services, or even healthcare providers themselves, through the additional burden of the second victim syndrome. Moreover, workload, bureaucracy, and profit-oriented economic policies are also important factors related to the perpetuation of dehumanization [24].

With regard to the demographic characteristics of the present study, the majority of the participants were female nurses, which is an expected result, given that nurses account for the largest professional group of health providers across the world [53]. Moreover, there is recent evidence that healthcare is executed in different ways, based on the gender of health professionals, since sex differences were found in pain management, with a preponderance of males in using some form of pain-reducing interventions, or in psychological preparedness for patient support [54,55,56]. It is noteworthy that the present study demonstrated significantly higher rates of self-dehumanization affecting male healthcare workers. This finding seems to be in accordance with a recent analysis, identifying gender differences in nurses’ values of life, and the male-nurse preponderance of misalignment between personal values and professional actions while other studies have reported higher psychoticism levels and depersonalization in males [57,58]. In a meta-analysis encompassing six concurrent studies, the findings indicated that the overall rate of burnout was notably higher among male nurses compared to their female counterparts during the COVID-19 pandemic. No differences were observed in emotional exhaustion or personal achievement between the two genders. However, the depersonalization scores were significantly higher in males [59].

Higher occurrence rates of mechanistic dehumanization were recorded among employees working in rotating shift patterns. Multiple studies on the topic address the impact of irregular shifts on the general well-being of nurses. The negative effects on metabolic and circadian rhythm stability, and lower proportions of work–life balance have recently been highlighted [57,60], while a recent study concluded that the root of hetero- or self-dehumanization lies in nurse dissatisfaction and negative work conditions, reflecting the quality of patient care [22].

In the present study, both types of hetero-dehumanization, as well as self-dehumanization in health professionals, were found to be significantly affected by certain personality traits, as expressed by the TIPI questionnaire. Multiple studies in the past advocate that social and emotional competencies appear to be crucial in most occupational service encounters, emphasizing their importance in fragile healthcare environments and the positive effects of authenticity on improving care quality and patient outcomes. Certain positive personality traits, such as agreeableness, extraversion, resilience, and compassion, have been identified as buffers against maladaptive interpersonal behaviors, while other researchers claim that these secure characteristics seem to motivate employees and enhance their work behavior, competence and well-being [34,36,61,62,63,64,65]. Furthermore, recent studies have extensively explored the negative associations of organizational dehumanization with deviant workplace behaviors and safety performance [66,67,68,69,70].

Empathic care was found to be a primary mediator and an important protective factor against all forms of dehumanization. In general, current medical education is mainly based on objectivity, emotional detachment, and on the notion of dehumanized healthcare which is currently being inculcated. Thus, modern medicine is impregnated with a widespread shortage of understanding for patients, and the formulation of “cognitive empathy” against “emotional empathy” has become predominant on healthcare grounds [71]. However, studies have shown that clinical empathy seems to improve both patients’ and healthcare providers’ well-being and also promotes better patient decision making and medical outcomes [71,72]. It is important that multiple medical schools currently pay more attention to compassion, as a step beyond empathy, or to prosocial behavior, as a means of strengthening the well-being and resilience for their healthcare students and shielding against burnout and compassion fatigue [73,74,75,76].

In the present study, burnout and secondary traumatic stress were found to be positively associated with all forms of dehumanization. Multiple studies highlight the association between dehumanization and burnout, along with the negative moderating role of moral injury, professional workload or stressors, and job dissatisfaction [67,77,78,79]. Moreover, a positive correlation between empathic engagement and burnout or secondary traumatic stress has been reported, with negative impacts on professional quality of life. Other studies have identified compassion fatigue as an important factor leading to a high turnover nursing workforce, while compassion satisfaction seems to be enhanced through robust education and supportive work environments [80,81]. Based on these clinical dimensions, current research addresses the need for illustrating strategies, in order to attenuate burnout and dehumanization at the interpersonal or intergroup level [38,82].

On the other side of the coin, dehumanization might also have certain protective functions that need to be addressed, since it might serve as a proactive emotional regulation strategy and a potential shield against medical burnout. Therefore, efforts are currently pointing towards a more flexible form of dehumanization, which might empower optimal decision making and performance in healthcare [23]. This entails a closed-loop and bidirectional flow between the “cognitive” and “emotional” elements of empathy. Furthermore, in the context of medical care, recent research has highlighted the importance of “embodiment”, a term referring to physicians’ personal qualities and authentic values, which seem to be the foundation of humanistic clinical practice. In fact, this patient-doctor intertwining might challenge and dramatize their “engaged curiosity”, and offer them the opportunity of illuminating their perspectives, obtaining self-awareness, and reshaping them as personalities towards a more authentic and empathic healthcare context [71,79,83]. Another recent review highlights that self-regulation of emotions during empathic engagement might be protective against burnout [84]. In line with this reasoning, the present report seeks to establish certain personality and empathic traits, acting as protective margins in the fragile balance between the different aspects of dehumanization [85,86].

Finally, the majority of the present study’s participants were reported as having good relationships with both colleagues (91.6%) and patients (93.6%). This suggests that despite the presence of dehumanization, there are strong interpersonal connections that can be leveraged, as protective measures against dehumanization and as a means of quality improvement in healthcare services. However, an important finding of the study is that a high percentage (17.8%) of healthcare workers attend psychotherapy sessions, which indicates that certain potential stressors, such as low income, workload or inherent challenges of the profession (illness, pain, death), might affect their mental health, impact their work performance, and force towards their psychological protection. The significance of this finding is probably highlighted by a number of studies that have shown the negative effects of income inequalities on overall mental health, depression, and psychosis in adults, which might probably explain the current expansion of dehumanization.

Interdisciplinary interventions about diverse cultural perceptions, social relationships, and institutional practices should further be investigated, to recognize early and confine the deleterious effects of dehumanization in the healthcare grounds [87,88].

### Limitations

The current study faces some limitations. One of the main limitations is that nearly two thirds of the respondents to this survey were nurses and the majority of these were females, reflecting the workforce. This is an expected result, given that most healthcare professionals in hospitals are nurses. Moreover, although the study sample is geographically and numerically representative, it was not selected through random sampling, while the cross-sectional design of the study limits the ability to establish causal relationships or evaluate changes differences among healthcare professionals. Finally, the present study also reports low-to-moderate Cronbach’s alphas of the TIPI questionnaire. Similarly to previous studies, reporting that the TIPI is generally not expected to reach the conventional standards of internal consistency, it should rather be conceptualized as a reflective measure, as already proposed in the current literature; therefore, the results based on this measure should be considered with caution [89,90].

## 5. Conclusions

This study underscores the critical need for a timely identification of all forms of dehumanization within healthcare settings and for addressing the vital role of empathy reinforcement among healthcare professionals as a buffer against deviant behaviors and poor-quality health services. Moreover, the present report seeks to establish certain personality traits, acting as protective margins in the fragile balance between the different aspects of dehumanization and burnout. It is essential for healthcare leadership to address the challenges faced by workers in this field, focusing on restoring their trust and meeting their needs. Authorities should prioritize assessing the risks faced by healthcare professionals and implement protective measures, while also developing and applying policies aimed at both prevention and active interventions. Implementing multi-tiered psychiatric support programs within hospitals, such as psychological counseling, debriefing after difficult situations (e.g., patient losses), and group psychotherapy, seem to be critical, in order to boost the mental health of workers. Finally, an additional important intervention might be the development of an educational program for medical and nursing students, aimed at fostering empathy, based on the principles of the Person-Centered Approach and Person-Centered Medicine. These initiatives might enhance the well-being of healthcare employees and ultimately improve the quality of patient care.

In summary, this study highlights the need for further investigation of dehumanization and its effects on healthcare, aiming to develop measures that will enhance the support and training of professionals, thereby improving the quality of care and the well-being of both patients and providers.

## Figures and Tables

**Table 1:** Demographic and other characteristics of the sample (N = 1150).

**Age** (years)		45.13 ± 10.25	
**Gender**		n	%
	Male	274	23.8
	Female	875	76.1
	Other	1	0.1
**Place of living**			
	Village	162	14.1
	Town (<150,000 citizens)	370	32.2
	City (>150,000 citizens)	618	53.7
**Family status**			
	Unmarried	395	34.3
	Married	652	56.7
	Divorced	88	7.7
	Widow/er	15	1.3
**Job**			
	Nurse	816	71.0
	Doctor	334	29.0
**Annual family income**			
	<20,000 €	972	84.5
	20–50,000 €	160	13.9
	50–80,000 €	13	1.1
	80–100,000 €	5	0.4
**Postgraduate studies (n = 251)**			
	Master	193	76.9
	PhD	58	23.1
**Work department**			
	Open department	560	48.7
	Closed department	589	51.2
	Laboratory	1	0.1
**Years of work**			
	From 6 months to 1 year	146	12.7
	From 2 years to 10 years	306	26.6
	From 11 years to 20 years	297	25.8
	From 21 years to 40 years	401	34.9
**Years of work in current department**			
	From 6 months to 1 year	263	22.9
	From 2 years to 10 years	478	41.6
	From 11 years to 20 years	286	24.9
	From 21 years to 40 years	123	10.7
**Working hours**			
	Circular timetable	877	76.3
	Morning/afternoon hours	273	23.7
**Working city**			
	Athens	179	15.6
	Thessaloniki	184	16.0
	Heraklion	346	30.1
	Other	441	38.4
**Relationship with colleagues**			
	Very good	693	60.3
	Good	360	31.3
	Typical	88	7.7
	Bad	2	0.2
	Very bad	7	0.6
**Relationship with patients (n = 1148)**			
	Very good	707	61.6
	Good	367	32.0
	Typical	70	6.1
	Bad	3	0.3
	Very bad	1	0.1
**Attending psychotherapy sessions**			
	Yes	205	17.8
	No	945	82.2
**If yes, for how long? (n = 205)**			
	6 months	123	60.0
	1 year	57	27.8
	2–5 years	16	7.8
	>5 years	9	4.4

Notes. Values are referred to absolute and relative frequencies (%) or means ± standard deviations (SD), minimum and maximum.

**Table 2:** Correlation coefficients of the hetero-dehumanization and self-dehumanization scale.

		Animalistic Hetero-Dehumanization	Mechanistic Hetero-Dehumanization	Self-Dehumanization Scale
**Age**		−0.033	−0.033	0.034
**Ten-Item Personality Inventory (TIPI)**				
Extraversion		0.017	0.052	−0.190 ***
Agreeableness		−0.216 ***	−0.182 ***	−0.233 ***
Conscientiousness		−0.085 **	−0.104 ***	−0.185 ***
Emotional Stability		−0.113 ***	−0.125 ***	−0.203 ***
Openness Experiences		−0.084 **	−0.081 **	−0.332 ***
**The Toronto Empathy Questionnaire (TEQ)**				
TEQ total		−0.294 ***	−0.241 ***	−0.455 ***
**Professional Quality of Life Scale (PROQOL)**				
Compassion		−0.225 ***	−0.286 ***	−0.370 ***
Burnout Scale		0.189 ***	0.197 ***	0.290 ***
Secondary Traumatic Stress		0.156 ***	0.078 **	0.256 ***
**Evaluation and Self-Report** **Questionnaire for the Quality of Provided Healthcare Services**				
Quality of Interaction		−0.188 ***	−0.283 ***	−0.198 ***
Long-term Utility from the Hospital		−0.183 ***	−0.223 ***	−0.227 ***
Immediate Perception of Quality		−0.115 ***	−0.133 ***	−0.201 ***
Satisfaction		−0.200 ***	−0.263 ***	−0.163 ***
Overall Perceived Quality		−0.142 ***	−0.169 ***	−0.215 ***

Notes. Values are referred to correlation coefficients Pearson. *** *p* < 0.001, ** *p* < 0.01.

**Table 3:** Demographic and other characteristics according to hetero-dehumanization and self-dehumanization scale.

		Animalistic Hetero-Dehumanization	Mechanistic Hetero-Dehumanization	Self-Dehumanization Scale
**Gender**				
	Male	2.90 ± 0.90	3.08 ± 0.96	3.64 ± 0.93
	Female	2.84 ± 0.87	3.07 ± 0.89	3.50 ± 0.94
*p*-value		0.357	0.799	**0.031**
**Job**				
	Nurse	2.87 ± 0.90	3.05 ± 0.89	3.53 ± 0.95
	Doctor	2.81 ± 0.84	3.11 ± 0.93	3.53 ± 0.92
*p*-value		0.321	0.376	0.938
**Work department**				
	Open department	2.84 ± 0.92	3.07 ± 0.91	3.52 ± 0.94
	Closed department	2.86 ± 0.84	3.07 ± 0.90	3.55 ± 0.94
*p*-value		0.661	0.876	0.500
**Working hours**				
	Circular timetable	2.86 ± 0.87	3.10 ± 0.91	3.54 ± 0.95
	Morning/afternoon hours	2.82 ± 0.92	2.96 ± 0.87	3.53 ± 0.92
*p*-value		0.492	**0.017**	0.880
**Attending psychotherapy sessions**				
	Yes	2.91 ± 0.95	3.18 ± 0.91	3.43 ± 1.00
	No	2.84 ± 0.86	3.05 ± 0.90	3.56 ± 0.93
*p*-value		0.277	0.058	0.095

Notes. Values are referred to means ± standard deviations (SD). *p*-value is computed using *t*-test. Statistically significant differences are highlighted in bold.

**Table 4:** Mediation analyses of Ten-Item Personality Inventory (TIPI) to hetero-dehumanization and self-dehumanization scale with The Toronto Empathy Questionnaire (TEQ) as mediator.

	Effect ^1^	Effect ^2^	Effect ^3^	Indirect Effect
Animalistic				
Extraversion	0.48 *** [0.18, 0.77]	−0.04 *** [−0.05, −0.03]	0.03 [−0.01, 0.07]	−0.02 *** [−0.03, −0.01]
Agreeableness	2.39 *** [2.03, 2.75]	−0.03 *** [−0.04, −0.02]	−0.11 *** [−0.16, −0.06]	−0.08 *** [−0.10, −0.06]
Conscientiousness	1.16 *** [0.75, 1.57]	−0.04 *** [−0.04, −0.03]	−0.04 [−0.09, 0.02]	−0.04 *** [−0.06, −0.02]
Emotional Stability	0.51 *** [0.20, 0.83]	−0.04 *** [−0.04, −0.03]	−0.06 *** [−0.10, −0.02]	−0.02 *** [−0.03, −0.01]
Openness Experiences	1.14 *** [0.78, 1.50]	−0.04 *** [−0.04, −0.03]	−0.03 [−0.07, 0.02]	−0.04 *** [−0.06, −0.03]
Mechanistic				
Extraversion	0.48 *** [0.18, 0.77]	−0.03 *** [−0.04, −0.03]	0.05 ** [0.01, 0.09]	−0.02 *** [−0.03, −0.01]
Agreeableness	2.39 *** [2.03, 2.75]	−0.03 *** [−0.03, −0.02]	−0.10 ** [−0.15, −0.04]	−0.06 *** [−0.09, −0.04]
Conscientiousness	1.16 *** [0.75, 1.57]	−0.03 *** [−0.04, −0.02]	−0.06 ** [−0.12, −0.01]	−0.04 *** [−0.05, −0.02]
Emotional Stability	0.51 *** [0.20, 0.83]	−0.03 *** [−0.04, −0.02]	−0.07 ** [−0.11, −0.03]	−0.02 *** [−0.03, −0.01]
Openness Experiences	1.14 *** [0.78, 1.50]	−0.03 *** [−0.04, −0.02]	−0.03 [−0.08, 0.02]	−0.04 *** [−0.05, −0.02]
Self-dehumanization				
Extraversion	0.48 *** [0.18, 0.77]	−0.06 *** [−0.07, −0.05]	−0.10 *** [−0.14, −0.07]	−0.03 *** [−0.05, −0.01]
Agreeableness	2.39 *** [2.03, 2.75]	−0.06 *** [−0.07, −0.05]	−0.08 *** [−0.13, −0.03]	−0.14 *** [−0.17, −0.11]
Conscientiousness	1.16 *** [0.75, 1.57]	−0.06 *** [−0.07, −0.05]	−0.11 *** [−0.16, −0.06]	−0.07 *** [−0.10, −0.04]
Emotional Stability	0.51 *** [0.20, 0.83]	−0.06 *** [−0.07, −0.05]	−0.12 *** [−0.16, −0.08]	−0.03 *** [−0.05, −0.01]
Openness Experiences	1.14 *** [0.78, 1.50]	−0.06 *** [−0.06, −0.05]	−0.22 *** [−0.27, −0.18]	−0.06 *** [−0.09, −0.04]

Notes. Values are referred to coefficients β and 95% Confidence Interval [95% CI]. *** *p* < 0.001, ** *p* < 0.01. Effect ^1^: Independent → Mediator; Effect ^2^: Mediator → Dependent; Effect ^3^: Independent → Dependent. Independent variables: scales of Ten-Item Personality Inventory (TIPI); dependent variables: scales of hetero-dehumanization and self-dehumanization scale; mediator: The Toronto Empathy Questionnaire (TEQ).

## Data Availability

The data that support the findings of this study are available from the corresponding author upon reasonable request.
